# Tanshinone IIA combined with adriamycin inhibited malignant biological behaviors of NSCLC A549 cell line in a synergistic way

**DOI:** 10.1186/s12885-016-2921-x

**Published:** 2016-11-18

**Authors:** Jun Xie, Jia-Hui Liu, Heng Liu, Xiao-Zhong Liao, Yuling Chen, Mei-Gui Lin, Yue-Yu Gu, Tao-Li Liu, Dong-Mei Wang, Hui Ge, Sui-Lin Mo

**Affiliations:** 1 The First Affiliated Hospital, Sun Yat-sen University, Guangzhou, 510080 People’s Republic of China; 2 School of Chinese Medicine, The University of Hong Kong, Hong Kong S.A.R., People’s Republic of China; 3 Kiang Wu Hospital, Macau S.A.R., People’s Republic of China; 4 Liwan District Shiweitang Street Community Health Service Center, Guangzhou, 510360 People’s Republic of China; 5 The Fifth Affiliated Hospital, Sun Yat-sen University, Zhuhai, 519000 People’s Republic of China; 6 School of Pharmaceutical Sciences, Sun Yat-sen University, Guangzhou Higher Education Mega Center, Guangzhou, 510006 People’s Republic of China

**Keywords:** NSCLC, Tanshinone IIA, Adriamycin, Synergistic effect, A549, VEGF/PI3K/Akt signal pathway

## Abstract

**Background:**

The study was designed to develop a platform to verify whether the extract of herbs combined with chemotherapy drugs play a synergistic role in anti-tumor effects, and to provide experimental evidence and theoretical reference for finding new effective sensitizers.

**Methods:**

Inhibition of tanshinone IIA and adriamycin on the proliferation of A549, PC9 and HLF cells were assessed by CCK8 assays. The combination index (CI) was calculated with the Chou-Talalay method, based on the median-effect principle. Migration and invasion ability of A549 cells were determined by wound healing assay and transwell assay. Flow cytometry was used to detect the cell apoptosis and the distribution of cell cycles. TUNEL staining was used to detect the apoptotic cells. Immunofluorescence staining was used to detect the expression of Cleaved Caspase-3. Western blotting was used to detect the proteins expression of relative apoptotic signal pathways. CDOCKER module in DS 2.5 was used to detect the binding modes of the drugs and the proteins.

**Results:**

Both tanshinone IIA and adriamycin could inhibit the growth of A549, PC9, and HLF cells in a dose- and time-dependent manner, while the proliferative inhibition effect of tanshinone IIA on cells was much weaker than that of adriamycin. Different from the cancer cells, HLF cells displayed a stronger sensitivity to adriamycin, and a weaker sensitivity to tanshinone IIA. When tanshinone IIA combined with adriamycin at a ratio of 20:1, they exhibited a synergistic anti-proliferation effect on A549 and PC9 cells, but not in HLF cells. Tanshinone IIA combined with adriamycin could synergistically inhibit migration, induce apoptosis and arrest cell cycle at the S and G2 phases in A549 cells. Both groups of the single drug treatment and the drug combination up-regulated the expressions of Cleaved Caspase-3 and Bax, but down-regulated the expressions of VEGF, VEGFR2, p-PI3K, p-Akt, Bcl-2, and Caspase-3 protein. Compared with the single drug treatment groups, the drug combination groups were more statistically significant. The molecular docking algorithms indicated that tanshinone IIA could be docked into the active sites of all the tested proteins with H-bond and aromatic interactions, compared with that of adriamycin.

**Conclusions:**

Tanshinone IIA can be developed as a novel agent in the postoperative adjuvant therapy combined with other anti-tumor agents, and improve the sensibility of chemotherapeutics for non-small cell lung cancer with fewer side effects. In addition, this experiment can not only provide a reference for the development of more effective anti-tumor medicine ingredients, but also build a platform for evaluating the anti-tumor effects of Chinese herbal medicines in combination with chemotherapy drugs.

**Electronic supplementary material:**

The online version of this article (doi:10.1186/s12885-016-2921-x) contains supplementary material, which is available to authorized users.

## Background

Lung cancer is a leading cause of cancer death worldwide, with a 5-year survival rate of 5–15% [1]. Non-small cell lung cancer (NSCLC), accounting for approximately 85% of all lung cancer cases, is the dominant type. Nowadays, platinum-based chemotherapy is considered the standard treatment for most advanced NSCLC patients. However, the tremendous side effects caused by chemotherapy severely impact the efficacy of treatments as well as the quality of life [2], indicating there is room for improvement in treatment methods [3, 4].

Adriamycin (ADM) has a broad anti-tumor effect, and is widely used in the treatment of various cancers. However, as other single agent treatment, it can cause bone marrow suppression, alopecia, nausea, and other adverse reactions. Long-term use of single agent may result in dose-dependent irreversible cardiomyopathy, causing severe cardiac toxicity and liver damage. The emergence of drug resistance and potential side effects highlight the major limitations for the single agent treatment in the clinical application [5]. In order to improve the anti-tumor effects and reduce the adverse reactions of chemotherapeutics, drug combination treatment is one of the solutions. Therefore, a search for novel strategies of combinational usage of agents to increase chemotherapeutic efficacy, and minimize associated toxicities to noncancerous tissues, should be at the forefront of oncology research [6].

Tanshinone IIA (1,6,6-trimethyl-6,7,8,9-tetrahydro-phenanthro [1,2-b] furan-10,11-dione), whose molecular formula is C_19_H_18_O_3_ and molecular mass is 294.344420 g/mol. Tanshinone IIA is one of the main fat-soluble compositions isolated from *Salvia miltiorrhiza*, that known as ‘Dan-Shen’ in traditional Chinese medicine [7]. The compound ID (CID) of tanshinone IIA in PubChem Compound is 164676.

The anti-tumor effects of tanshinone IIA on a broad of cancer cells have been tested in vitro, including lung [8], liver [9], stomach [10] and pancreatic cancer cells [11]. Our previous studies showed that tanshinone IIA inhibited the growth of NSCLC A549 cell line by decreasing VEGF/VEGFR2 expression [12]. It has been documented that the combination of tanshinone IIA and ADM not only could exhibit a synergistic effect on HepG2, but also improve the cytotoxicity of ADM with less cardiotoxicity [9]. Additionally, it has been found that tanshinone IIA could protect cardiomyocytes from ADM-induced apoptosis in part through Akt-signaling pathways [13]. These studies indicate that tanshinone IIA may serve as an effective adjunctive reagent in the treatment of NSCLC. However, the effect of tanshinone IIA in combination with ADM on NSCLC cells remains unclear.

In this study, we tried to investigate whether tanshinone IIA and ADM may present a synergistic anti-tumor effect on human NSCLC cell lines A549 and PC9. Furthermore, the underlying molecular mechanisms of the combination of both reagents were investigated as well. The evaluation methods of synergistic effect of agents, virtual screen and confirmed strategies for the involved target proteins were applied in our study, which could be a novel strategy for the evaluation and investigation of combination and interaction of anti-tumor drugs.

## Methods

### Cell lines, culture condition and reagents

**Fig. 1:**
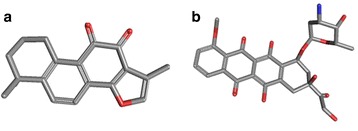
The three-dimensional (3D) structure of  tanshinone IIA (**a**) and ADM (**b**) (from PubChem compound http://pubchem.ncbi.nlm.nih.gov/)

The human NSCLC cell line A549 and PC9, and the Human Lung Fibroblast (HLF) cell line were supplied by the Cell Bank of the Chinese Academy of Sciences (Shanghai, China) and cultured in RPMI1640 (Gibco, Carlsbad, CA, USA), supplemented with 10% fetal bovine serum (Gibco, Carlsbad, CA, USA) in a humidified incubator at 37 °C, 5% CO_2_ atmosphere. Tanshinone IIA (Fig. 1a) was purchased from Sigma-Aldrich (St. Louis, MO, U.S.A.) and prepared as a 10 mM stock solution in dimethylsulfoxide (DMSO) (St. Louis, MO, USA). The solution was serially diluted in a RPMI 1640 medium immediately prior to the experiments. ADM (Fig. 1b) was purchased from Sigma-Aldrich and prepared as a 10 mM stock solution in normal saline (NS) which was serially diluted in RPMI 1640 medium immediately prior to experiments. Pancreatin, penicillin and streptomycin were purchased from Gibco (Invitrogen Life Technologies, Carlsbad, CA, USA). All the reagents were of analytical grade.

### Cell viability assay

Cell proliferation was evaluated using the CCK8 (Dojindo Laboratories, 119 Kumamoto, Japan) according to manufacturer’s instructions. Briefly, A549, PC9 or HLF Cells (6 × 10^3^/90 μL/well) were plated into 96-well plates in triplicate and cultured for 24 h before onset of treatment. Then cells were treated with ADM, tanshinone IIA and combination of both drugs at a fixed molar ratio over a broad dose range to establish growth curves for 48 h. After that, cells were incubated for an additional 2 h with CCK-8 reagent (100 μl/mL medium). The absorbance was determined at 450 nm wavelength with a reference wavelength of 630 nm using a microplate reader (BioTek, Winooski, 126 VT, USA). The proliferative inhibition rate was measured using the Optical Density and calculated using the formula: proliferative inhibition rate = (1-treatment group/control group) × 100%. The IC_50_ (50% inhibitory concentration) value was calculated by nonlinear regression analysis using GraphPad Prism software (San Diego, CA, USA).

### Synergy determination

The isobologram analysis for the combination study was based upon the Chou-Talalay method to determine combination indices (CI). The data obtained with the CCK8 assay was normalized to the vehicle control and expressed as % viability. Then, the data was converted to Fraction affected (Fa; range 0–1; where Fa = 0 represents 100% viability and Fa = 1 represents 0% viability) and analyzed with the CompuSyn™ program (Biosoft, Ferguson, MO) based upon the Chou and Talalay median effect principle [14, 15]. The CI values reflect the ways of interaction between two drugs. CI < 1 indicates synergism, CI = 1 indicates an additive effect, and CI > 1 indicates antagonism [16].

### Wound healing assay

A549 cells (1 × 10^6^/1 mL/well) were plated in 6-well plates and allowed to adhere for 24 h. Confluent monolayer cells were scratched by a 200 μL pipette tip and then washed three times with 1 × PBS to clear cell debris and suspension cells. Fresh serum-free medium with different drug treatments were added, and the cells were allowed to close the wound for 48 h. Photographs (magnification, ×100) were taken at 0 h and 48 h at the same position of the wound. The migration distance was calculated by the change in wound size during the 48 h period using Adobe Photoshop CS6 software.

### Transwell assay

A549 cells (5 × 10^4^) were resuspended in 200 μl of serum-free medium containing different drug treatments and seeded on the top chamber of the 8 μm pore, 6.5 mm polycarbonate transwell filters (Corning, NY, USA), whose inserts were coated with a thin layer of 0.25 mg/ml Matrigel Basement Membrane Matrix (BD Biosciences, Bedford, MA). The full medium (600 μl) containing 10% FBS was added to the bottom chamber. The cells were allowed to migrate through the filters for 48 h at 37 °C in a humidified incubator with 5% CO_2_. The cells attached to the lower surface of membrane were fixed in 4% paraformaldehyde at room temperature for 30 min and stained with 0.5% crystal violet. The cells on the upper surface of the filters were removed by wiping with a cotton swab. The number of stained cells on the lower surface of the filters was counted under the microscope (magnification, ×100). A total of 5 fields were counted for each transwell filter.

### Flow cytometric cell cycle analysis

After incubation at 37 °C in an atmosphere of 5% CO_2_ for 48 h, the treated cells were detached by trypsinization, collected, washed twice with cold PBS and fixed in 5 mL 75% cold ethanol at 4 °C for 24 h. The cells were again washed twice with PBS and incubated with 500 μl RNase (50 μg/mL) for 30 min at 37 °C, and then labeled with propidium iodide (PI, 0.1 mg/mL) then incubated at room temperature in the dark for 30 min prior to analysis. For each measurement, at least 20,000 cells were counted. Cell cycle analysis was performed by analyzing PI staining levels by flow cytometry (Beckman Coulter, USA). Data was analyzed using ModFit (Verity Software House, Inc, Topsham, ME).

### Flow cytometric apoptosis assay

Cell apoptosis was determined by PI and Annexin V-FITC staining (KeyGEN Biotech, Nanjing, China). In brief, the treated cells were incubated for 48 h, washed twice with ice-cold PBS, the collected cells were then resuspended in 200 μl of binding buffer and incubated with 5 μl each of Annexin V-FITC and PI for 15 min in the dark at room temperature, according to the manufacturer’s instructions. The cells were analyzed immediately after staining, using a FACScan flow cytometer (Becton-Dickinson). For each measurement, at least 20,000 cells were counted.

### TUNEL assay

Apoptosis was detected using the In Situ Cell Death Detection Kit (Roche Molecular Bioscience, Mannheim, Germany) following manufacturer’s instructions. Apoptotic cells were imaged using a fluorescence microscope (Olympus, Tokyo, Japan). For each sample, three photomicrographs of random fields were taken at 400× magnification, and cells were scored as apoptotic or viable and counted. The percentage of apoptotic cells was determined by counting the TUNEL-positive cells and dividing the number by the total number of cells.

### Immunofluorescence assay

Immunofluorescence assay was applied to detect the expression of Cleaved Caspase-3. The treated cells were washed with PBS and then fixed with 4% paraformaldehyde for 15 min at room temperature. Permeabilization was done with 0.3% Triton X-100 for 30 min and then blocked with 5% normal FBS for 1 h at room temperature. After that cells were incubated overnight at 4 °C with anti-Cleaved Caspase-3 (1:200, Cell Signaling Technology, Beverly, MA) primary antibody. Secondary anti-mouse (1:500, Alexafluor488, Invitrogen, Carlsbad, USA) antibody was added for 1 h at room temperature in the dark. After washing with PBS three times, the coverslips were mounted on slides by using mounting medium containing DAPI (Invitrogen) and observed using a fluorescence microscope (Olympus, Tokyo, Japan) (magnification, ×400).

### Western blotting analysis

Western blotting analysis was applied for the re-confirm via molecular biological method. All the selected proteins extracts of each group cells were resolved by 10% SDS-PAGE and transferred on PVDF (Millipore, Bedford, MA, USA) membranes. After blocking, the PVDF membranes were washed four times for 15 min with TBST at room temperature and incubated with primary antibodies. The following primary antibodies were used: anti-Bax, anti-Bcl-2, anti-Caspase-3, anti-Akt, anti-phospho-Akt, anti-PI3K, anti-phospho-PI3K (all 1:1000; Cell Signaling Technology, Danvers, MA, USA), anti-VEGF (1:1000; Abcam, Cambridge, MA, USA), anti-Cleaved Caspase-3 (1:500; Cell Signaling Technology, Danvers, MA, USA), anti-VEGFR2, (1:200; Cell Signaling Technology, Danvers, MA, USA) and anti-GAPDH (1:2000; Cell Signaling Technology, Beverly, MA). Following extensive washing, membranes were incubated with secondary horseradish peroxidase (HRP)-conjugated secondary antibodies (1:1000; Cell Signaling Technology, Danvers, MA, USA) for 1 h. After washing 4 times for 15 min with TBST at room temperature once more, the immunoreactivity was visualized by enhanced chemiluminescence (ECL kit, Millipore, Billerica, MA, USA), and membranes were exposed to KodakXAR-5 films (Sigma-Aldrich). Relative optical density (ROD, ratio to GAPDH) of each blot band was quantified by using National Institutes of Health (NIH) image software (Image J 1.36b).

### Molecular docking algorithm

To predict the possible interaction of small molecules and the selected proteins, Discovery Studio (DS) 2.5 (Accelrys Software Inc, San Diego, CA) was applied to the molecular docking algorithm in this study. The calculation of root mean square deviation (RMSD) was carried out for the validation of the veracity for the selection of molecular docking modules in DS 2.5. The three-dimensional (3D) crystal structures of proteins were selected from PDB (http://www.rcsb.org/pdb/).The 3D structure of tanshinone IIA was downloaded from The PubChem Project (http://pubchem.ncbi.nlm.nih.gov/) with a CID of 164676. The DS 2.5 was run on a localhost9943 server. The docking procedure includes the following steps. Firstly, the water molecules in the proteins were removed and the hydrogen atoms were added to the proteins. Secondly, small molecules and selected proteins were refined with CHARMM. Thirdly, the active sites of proteins were defined by two methods: according to internal ligand’s binding site and automatically with DS 2.5. Lastly, small molecules were docked into the active sites of the proteins with the appropriate parameter settings. Through a series of algorithms, 10 different orientations were randomly generated. Each orientation was subjected to simulated annealing molecular dynamics simulation. ADM simulation was run consisting of a heating phase from 300 to 700 K with 2000 steps, followed by a cooling phase back to 300 K with 5000 steps. The energy threshold for Van der Waals force was set at 300 K. We further refined the simulation result by running a short energy minimization, consisting of 50 steps of steepest descent followed by up to 200 steps of conjugate gradient using an energy tolerance of 0.001 kcal/mol.

### Statistical analysis

All experiments were performed in triplicate and repeated at least three times, a representative experiment was selected for the figures. Data was presented as mean value ± standard error and was analyzed using SPSS 15.0 software by one-way ANOVA with Dunnett’s post hoc test and Turkey’s post hoc test for multi-group comparisons (except the IC_50_ values which were calculated by nonlinear regression analysis using GraphPad Prism software.). Student’s *t*-test was used for paired data. A *p* value of 0.05 or less was considered as significant. The drug interactions were assessed using multiple effect analysis based on the Chou-Talalay method.

## Results

### Co-treatment of tanshinone IIA and ADM synergistically decreased cell viability of A549 and PC9 cells

**Fig. 2:**
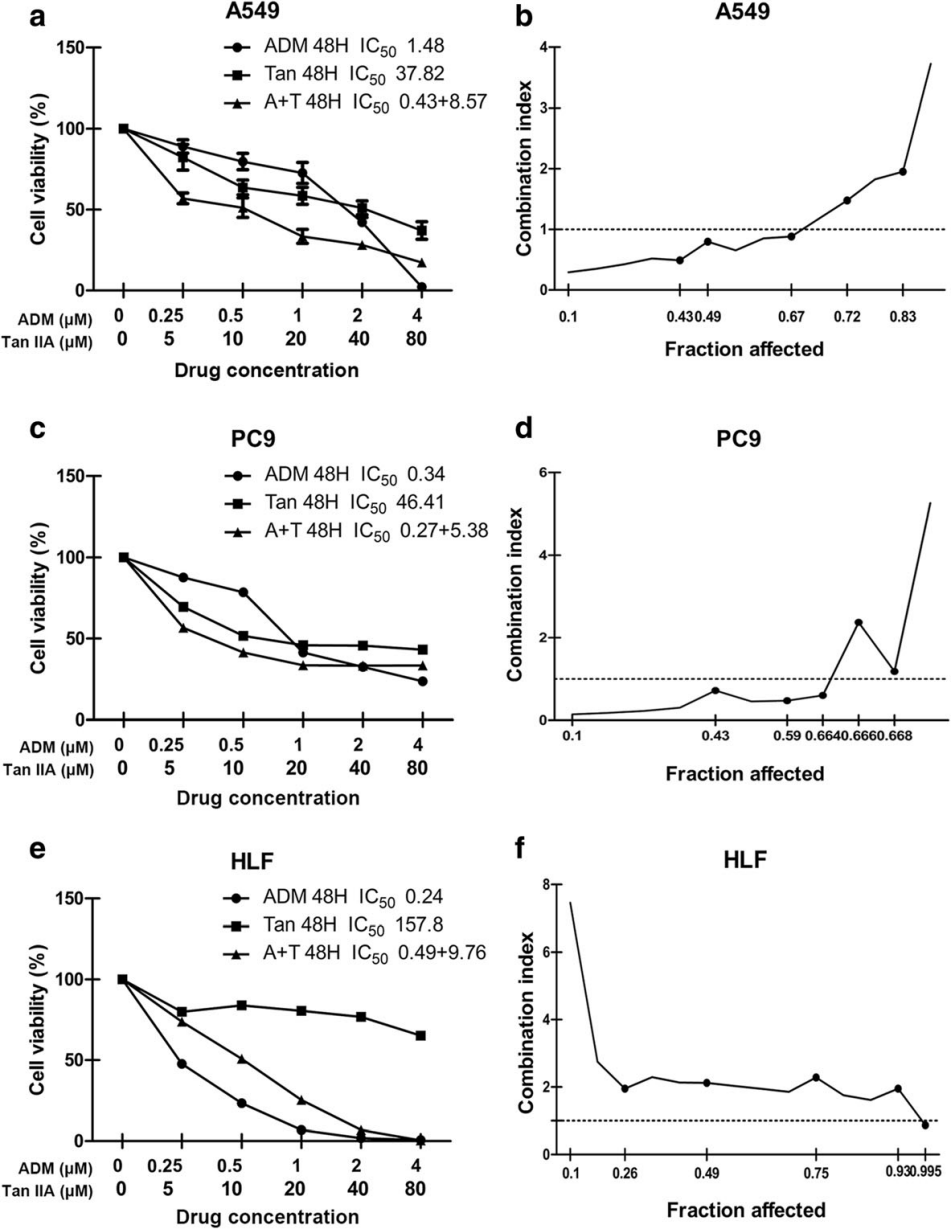
The proliferative inhibition assay of tanshinone IIA, ADM and tanshinone IIA in combination with ADM on A549, PC9, and HLF cell lines. Cells were exposed to various concentrations of tanshinone IIA and ADM alone or in combination at 20:1 molar ratio (tanshinone IIA: ADM) for 48 h. Cell viability curves were plotted as viable cell percentage based on the CCK8 assay (**a**, **c**, **e**). The synergistic effects between drugs were shown as Fa-CI plots calculated with the calcusyn™ software (**b**, **d**, **f**). Each plot (**a**, **c**, **e**) shows the average proliferative inhibition rate of three experiments with triplicate wells. (n = 3, mean ± SD) *P < 0.05, **P < 0.01, or ***P < 0.001 versus the vehicle control

As shown in Fig. 2 and Additional file 1, both ADM and tanshinone IIA inhibited the proliferation of the tested cell lines in a time- and dose-dependent manner, with HLF cells showing a lowest IC_50_ value of ADM and a highest IC_50_ value of tanshinone IIA among the tested cells. These data hinted that HLF cells displayed a stronger sensitivity to ADM, and a weaker sensitivity to tanshinone IIA, compared with the NSCLC A549 cell line and the NSCLC PC9 cell line.

Guided by the IC_50_ values determined for the single drugs, the combinations of the ADM and tanshinone IIA were evaluated at the 1:20 (ADM: tanshinone IIA) fixed molar ratio for 48 h. Compared to any individual drug, drug combination exerted a much stronger inhibition of cells growth, except HLF cells. In A549 cells, drug combination treatment had a synergistic inhibitory effect (CI <1) when Fa value was ≤0.67 (Fig. 2b). The synergism of drug combination treatment (CI <1) was observed when Fa value was ≤0.664 in PC9 cells (Fig. 2d). For HLF cells, ADM combined with tanshinone IIA induced significant antagonistic growth inhibition (CI>1) when Fa value was ≤0.99 (Fig. 2f) . The summary of CI value and the concentration of the separate drugs in combination at 50% Fa were shown in Table 1.

**Table 1:** Summary of CI value and the concentration of the separate drugs in combination at 50% Fa

Drug combination		Fa = 0.5		
		A549	PC9	HLF
Tan IIA + ADM	CI	0.654 ± 0.1392	0.456 ± 0.285	2.028 ± 0.3486
	Regimen			
	Tan IIA (μM)	8.57470	5.37651	9.76190
	ADM (μM)	0.42873	0.26883	0.48809

### Co-treatment of tanshinone IIA and ADM synergistically inhibited migration and invasion of A549 cells

**Fig. 3:**
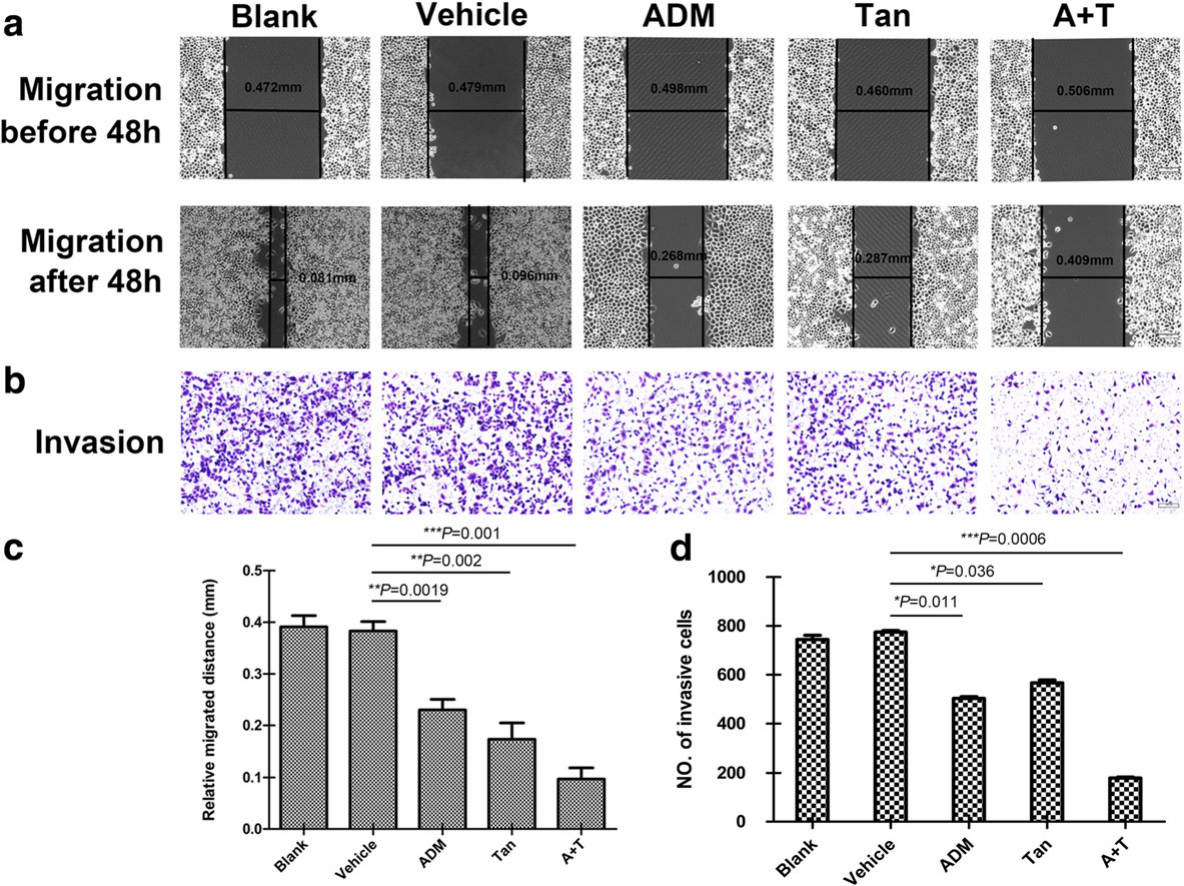
Tanshinone IIA and ADM inhibited migration and invasion of A549 cells. Representative images of wound healing assay (**a**) and transwell assay (**b**) after 48 h treatment with 36 μM of tanshinone IIA (48 h IC_50_ value) and 1.5 μM of ADM (48 h IC_50_ value) alone or in combination. Bar graphs represent the average migration distance (**c**) and the number of stained cells (**d**) respectively, which were calculated from the three independent experiments with ten fields counted per experiment. Data are presented as the means ± SD of three independent experiments. *P < 0.05, **P < 0.01, or ***P < 0.001 versus the vehicle control. (magnification, ×100. Scale bars, 100 μm)

Since A549 cell line is broadly used in lung cancer research area, we choose it as the further research object. To identify a combination that achieved maximal biological function, the migration and invasion ability in A549 cells were investigated by wound healing assay and transwell assay. Figure 3 showed that the migration distances and the invasive cell numbers were significantly decreased after 48 h drug treatment. Meanwhile, the combined simultaneous treatment showed the least migration distance and the invasive cell number in the results.

### Co-treatment of tanshinone IIA and ADM arrested cell cycle of A549 cells

**Fig. 4:**
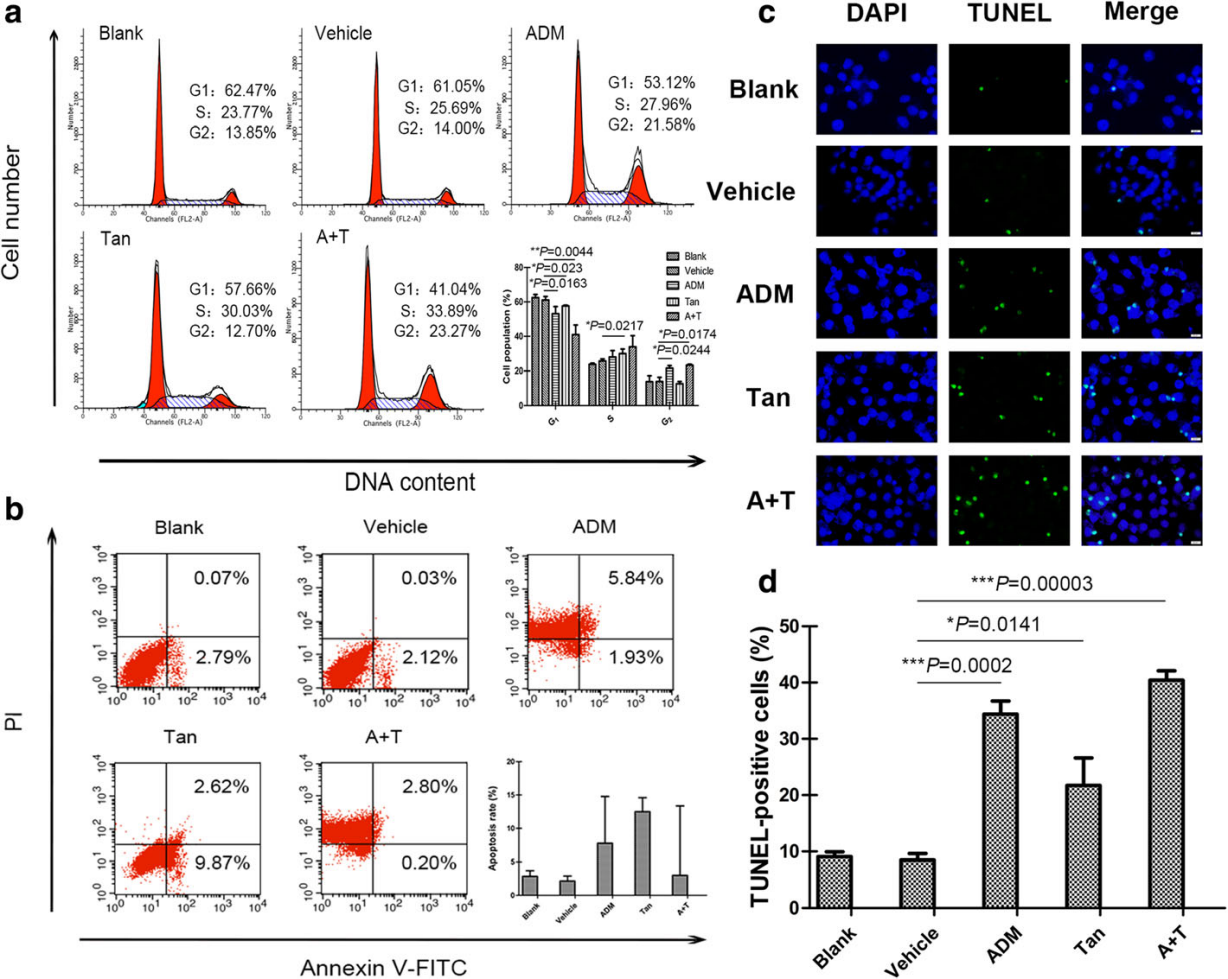
Effect of tanshinone IIA and ADM alone and in combination on the cell cycle arrest and apoptosis induction in A549 cells. The cell cycle distributions after 48 h treatment with 36 μM of tanshinone IIA and 1.5 μM of ADM alone or in combination ﻿(**a**). The apoptosis rate after 48 h treatment with 36 μM of tanshinone IIA and 1.5 μM of ADM alone or in combination (**b**). Representative photographs of TUNEL staining cells in various groups ﻿(**c**). Histogram of quantification of TUNEL-positive cells was shown with the percentage of TUNEL-positive nuclei (*green*) relative to DAPI-positive total nuclei (*blue*) (**d**). All data represent the mean ± SD of three independent experiments. *P < 0.05, **P < 0.01, or ***P < 0.001 versus the vehicle control

After verifying the anti-proliferation effect of tanshinone IIA and ADM, the distribution of cell cycles were detected by Flow cytometry. As shown in Fig. 4a, the cell population in G1 phase was decreased in both single drug treatment groups and drug combination groups, with the latter showing more deduction. At the same time, tanshinone IIA group increased the S phase cell population, the ADM and drug combination groups increased the G2 phase cell percentage in comparison with the untreated cells.

### Co-treatment of tanshinone IIA and ADM induced apoptosis of A549 cells

Then, we detect the cell apoptosis via Flow cytometry. Both single drug treatment and drug combination increased the proportion of early (dots in the lower right quadrant) and late apoptosis (dots in the upper right quadrant) in A549 cells (Fig. 4b). However, there was no statistical significance among the tested groups. TUNEL assays were performed to detect the DNA fragmentation in A549 cells after different drug treatments. The presence of TUNEL-positive cells (stained green), showing occurrences of DNA strand breaks, also indicates apoptosis in cells. Quantification revealed that all the tanshinone IIA groups, ADM groups, and drug combination groups increased the TUNEL-positive cells (Fig. 4d). Taken together, co-treatment more potently induced apoptosis compared with single treatment in A549 ﻿cells.

### Co-treatment of tanshinone IIA and ADM decreased the activity of VEGF/PI3K/Akt signaling pathway in A549 cells

**Fig. 5:**
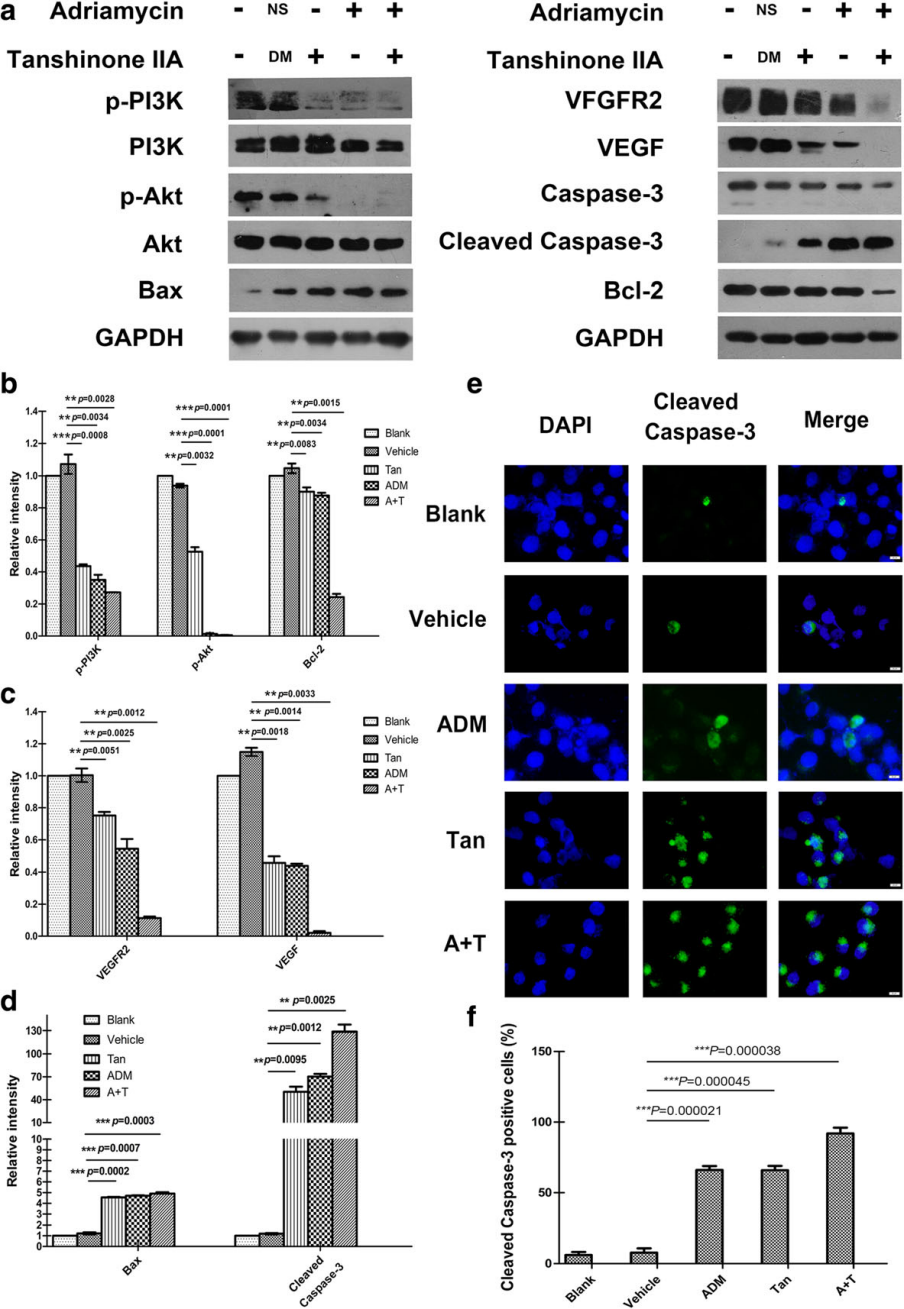
Effect of tanshinone IIA and ADM on the inhibition of VEGF/PI3K/Akt signaling pathway in A549 cells. Figures are the expression levels of VEGF, VEGFR2, PI3K, p-PI3K, Akt, p-Akt, Bcl-2, Bax, Caspase-3, Cleaved Caspase-3, and GAPDH after 48 h treatment with 36 μM of tanshinone IIA and 1.5 μM of ADM alone or in combination (**a**). The statistical histogram shows the Relative optical density of the tested proteins by Image J (**b**, **c**, **d**). Representative images show the immunofluorescence detection of Cleaved Caspase-3. Cells were stained with an antibody that can recognize Cleaved Caspase-3 (*green*), and then stained with DAPI (*blue*) to visualize nuclei (**e**). The statistical analysis of relative fluorescence intensity shows the expression of Cleaved Caspase-3 in A549 cells (**f**). Data are presented as the means ± SD of three independent experiments. *P < 0.05, **P < 0.01, or ***P < 0.001 versus the vehicle control. (magnification, ×400, Scale bars, 50 μm)

In order to explore the involved signal pathway, we performed western blotting to measure the levels of VEGF, VEGFR2, PI3K, p-PI3K, Akt, p-Akt, Bcl-2, Bax, Caspase-3, and Cleaved Caspase-3 upon single drug treatment groups and drug combination groups in A549 cells. Results revealed that both single drug treatment and drug combination up-regulated Bax, Cleaved Caspase-3 expression levels, but down-regulated VEGF, VEGFR2, Bcl-2, Caspase-3, p-Akt, and p-PI3K expression levels, with the total Akt, PI3K, and GAPDH levels remaining the same (Fig. 5a, b, c,﻿ d). Immunofluorescence assays were performed and the results revealed that Cleaved Caspase-3 level was consistently elevated by both single drug treatment and drug combination treatment in A549 cells (Fig. 5e, f). The effect of drug combination groups showed significant difference compared with single drug treatment groups in both immunofluorescence assay and western blot assay.

### Molecular docking algorithm

Molecular docking algorithm was applied to predict the possible interaction of small molecules and the selected proteins. First of all, as shown in Table 2, the RMSD of Akt2 (PDB-ID: 2JDR), Bcl2 (PDB-ID: 4IEH), PI3K (PDB-ID: 4J6I), and VEGFR-2 (3VHE) were 0.6208 Å, 1.370 Å, 0.8333 Å, and 0.3568 Å, respectively, when CDOCKER module in DS 2.5 was applied in the algorithms. It presented the veracity of CDOCKER in the study.

**Table 2:** The validation of molecular docking algorithm (RMSD)

Protein	CDOCKER RMSD (Å)	Libdock RMSD (Å)
2JDR	0.6208	4.6929
4IEH	1.370	8.19049
4J6I	0.8333	4.1504
3VHE	0.3568	5.76

**Fig. 6:**
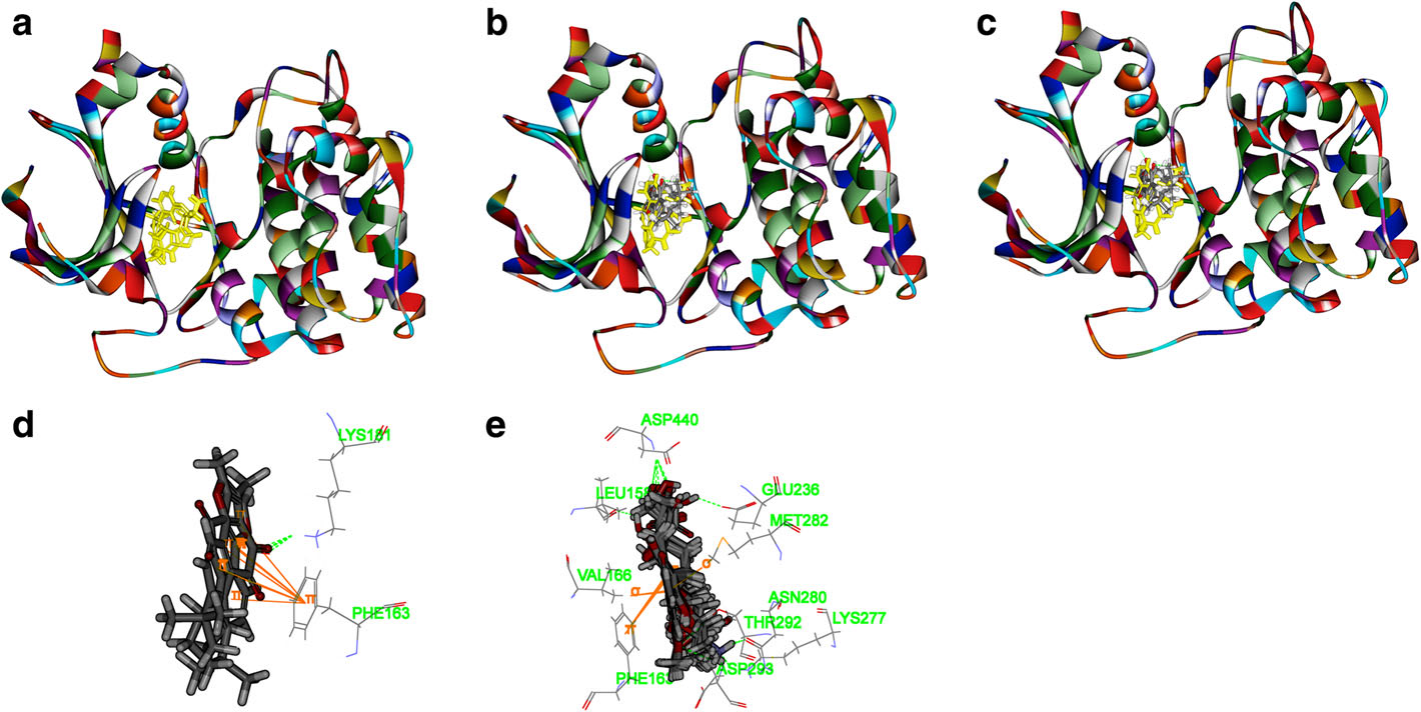
The structure of Akt2 (2JDR) and binding site: Fig. 6**a** shows the 3D structure of crystal structure of human Akt2 with an endogenous ligand (PDBID:2JDR). The solid ribbon is the 3D structure of crystal structure of human Akt2 with a 2.3 Å resolution. In the centre of 2JDR is an endogenous ligand (*yellow*) bound in the interface. Figure 6**b** shows ten poses of tanshinone IIA docked into the endogenous ligand’s (*yellow*) active site of 2JDR. Figure 6**c** shows ten poses of ADM docked into the endogenous ligand’s (*yellow*) active site of 2JDR. Figure 6**d** shows the binding model of tanshinone IIA in Akt2: at least two residues involved in the interactions in ten random poses, one is Lys181 (H-bond), another is Phe163 (aromatic interactions). Fig. 6**e** shows the binding model of ADM in Akt2: at least ten residues involved in the interactions in ten random poses, Leu158, Glu236, Lys277, Asp440, Asn280, Thr292 and Asp293 (H-bonds), Phe163, Val166, and Met282 (aromatic interactions).

**Fig. 7:**
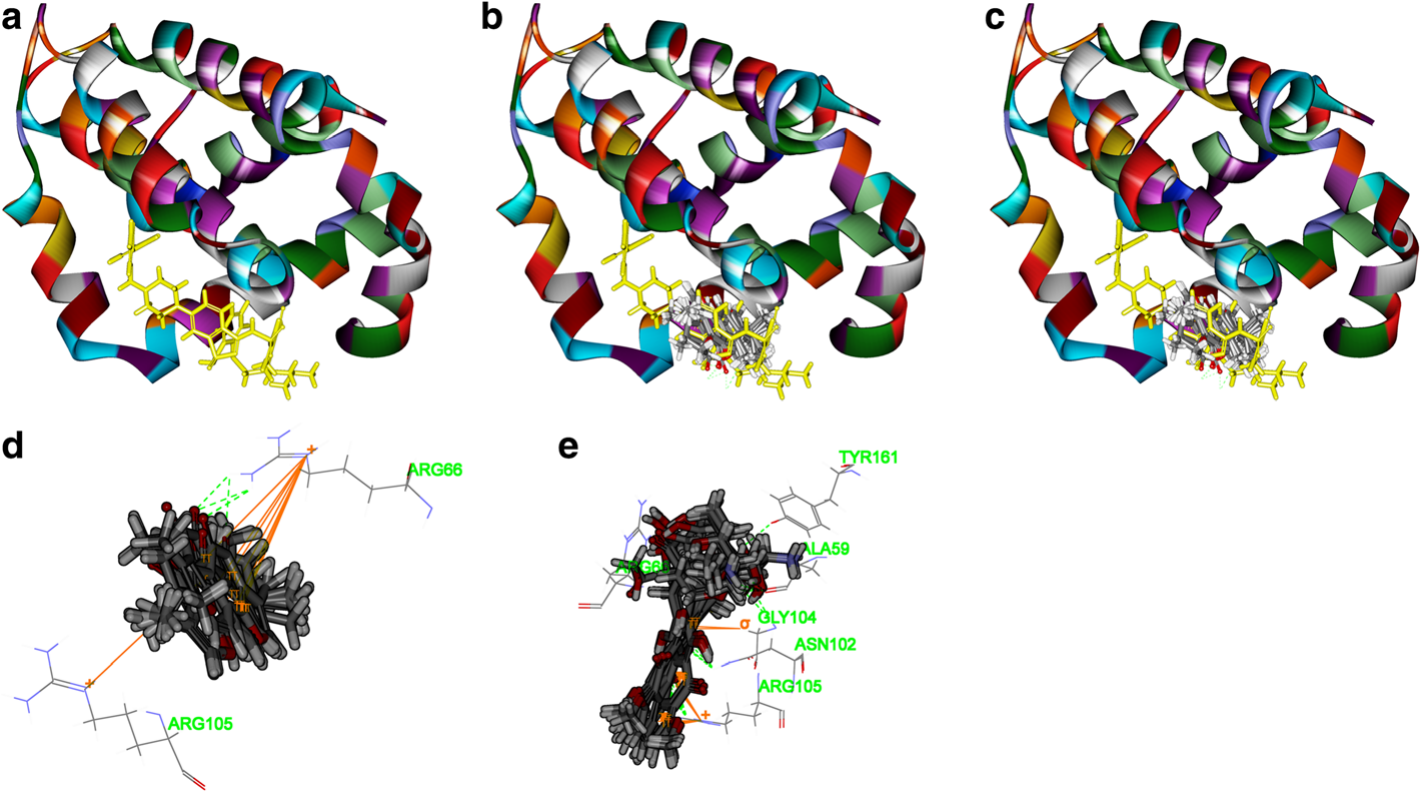
The structure of Bcl2 (4IEH) and binding site: Fig. 7**a** shows the 3D structure of crystal structure of human Bcl2 with an endogenous ligand (PDBID: 4IEH). The solid ribbon is the 3D structure of crystal structure of human Bcl2 with a 2.1 Å resolution. In the centre of 4IEH is an endogenous ligand (*yellow*) bound in the interface. Figure 7**b** shows ten poses of tanshinone IIA docked into the endogenous ligand’s (*yellow*) active site of 4IEH. Figure 7**c** shows ten poses of ADM docked into the endogenous ligand’s (*yellow*) active site. Figure 8**d** shows the binding model of tanshinone IIA in Bcl-2: at least two residues involved in the interactions in ten random poses, Arg105 (H-bonds), and Arg66 (aromatic interactions). Figure 8**e** shows the binding model of ADM in Bcl-2: at least six residues involved in the interactions in ten random poses, Ala59, Arg66, Asn102, and Try161 (H-bonds), Gly104, and Arg105 (H-bonds plus aromatic interactions)

**Fig. 8:**
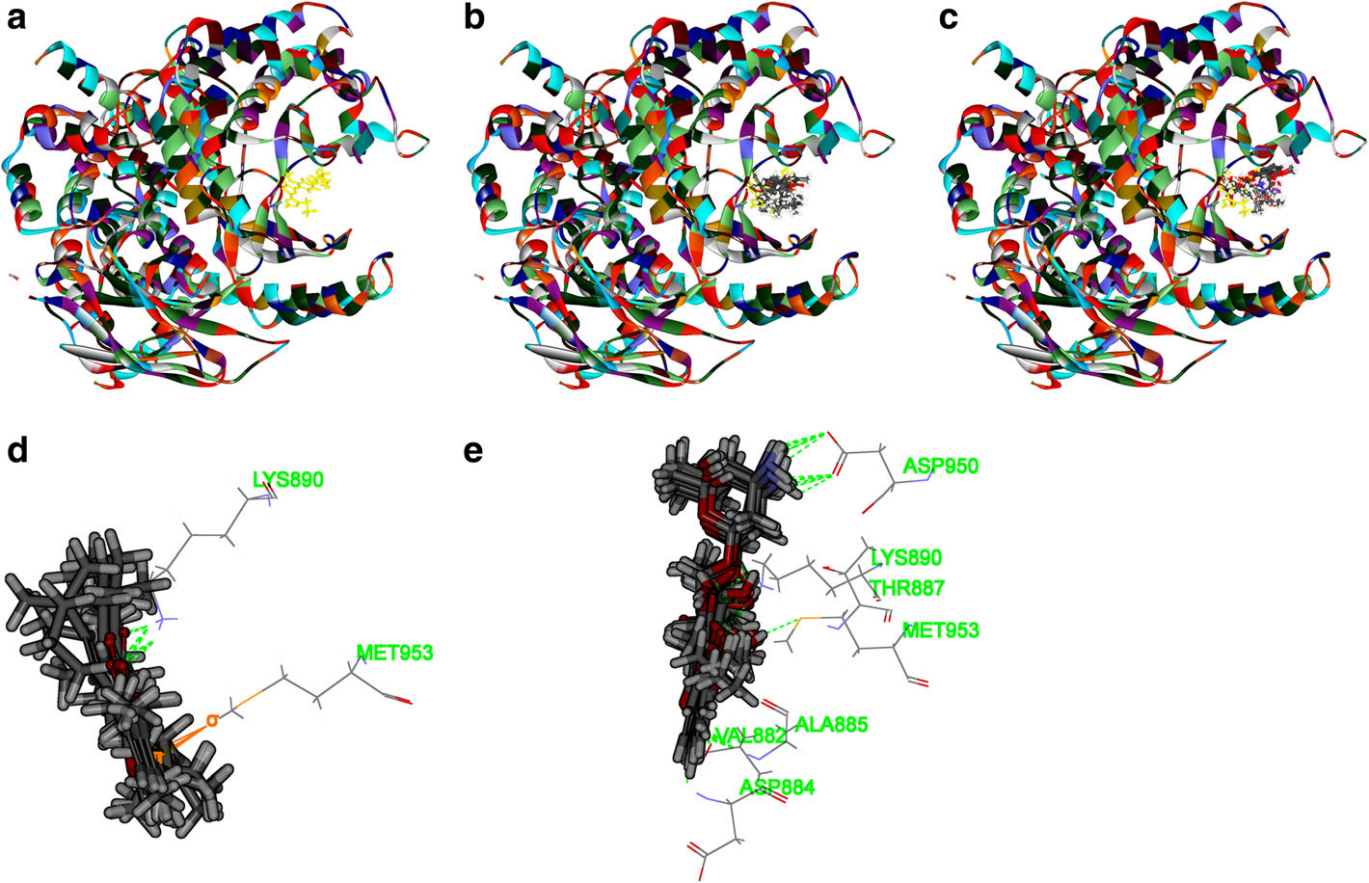
The structure of PI3K (﻿4J6I) and binding site: Fig. 8**a** shows the 3D structure of crystal structure of human PI3K with an endogenous ligand (PDBID: 4J6I). The solid ribbon is the 3D structure of crystal structure of human PI3K with a 2.9 Å resolution. In the centre of 4J6I is an endogenous ligand (*yellow*) bound in the interface. Figure 8**b** shows ten poses of tanshinone IIA docked into the endogenous ligand’s (*yellow*) active site of 4J6I. Figure 8**c** shows ten poses of ADM docked into the ligand’s (*yellow*) active site of 4J6I. Figure 8**d** shows the binding model of tanshinone IIA in 4J6I: at least two residues involved in the interactions in ten random poses, one is Lys890 (H-bond), another is Met953 (aromatic interaction). Figure 8**e** shows the binding model of ADM in 4J6I: at least seven residues involved in the interactions, Val882, Ala885, Asp884, Thr887, Lys890, Asp950, and Met953 (H-bonds)

**Fig. 9:**
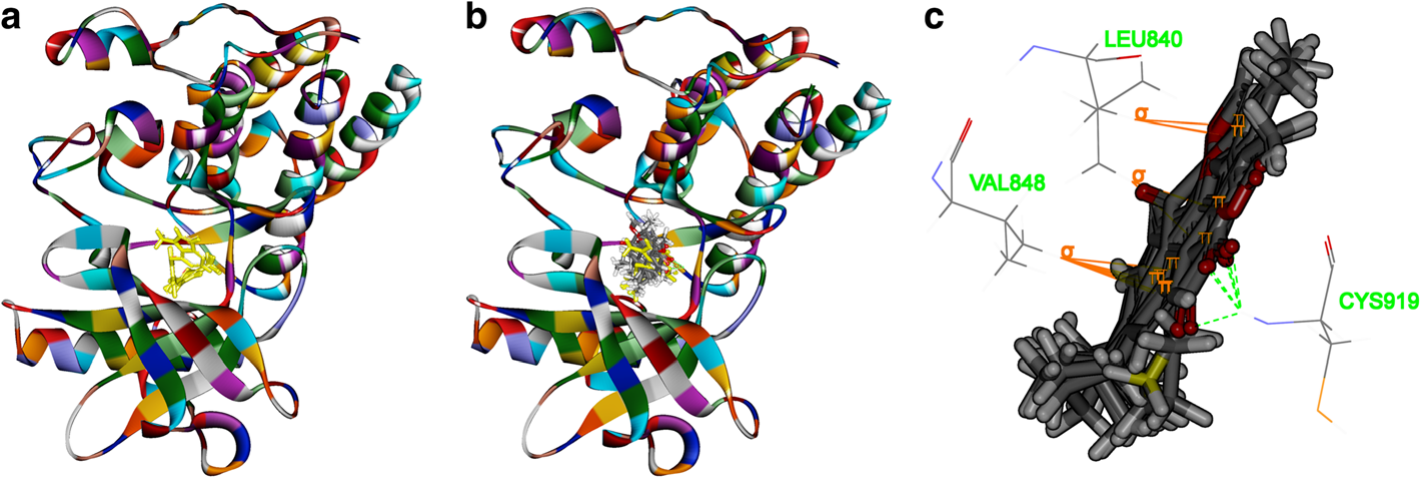
The structure of VEGFR-2 (3VHE) and binding site: Fig. 9**a** shows the 3D structure of crystal structure of human VEGFR-2 (PDBID: 3VHE). The solid ribbon is the 3D structure of crystal structure of 3VHE with a 1.55 Å resolution. In the center of 3VHE is a kinase domain inhibitor bound in the interface. Figure 9**b** shows ten poses of tanshinone IIA docked into the endogenous ligand’s (*yellow*) active site of 3VHE. Figure 9**c** shows the binding model of tanshinone IIA in 3VHE: at least three residues involved in the interactions, Cys919 (H-bond), Leu840, and Val848 (aromatic interactions)

Secondly, as shown in Figs. 6, 7, 8 and 9, tanshinone IIA could be docked into active sites of all the proteins with individual binding modes, when compared with ADM. Tanshinone IIA could form H-bonds with Lys181 and aromatic interactions with Phe163 in the endogenous ligand’s active site of Akt2 (PDB-ID: 2JDR), while ADM formed H-bonds with Leu158, Glu236, Lys277, Asp440, Asn280, Thr292, and Asp293, aromatic interactions with Phe163, Val166, and Met282 (Fig. 6). Tanshinone IIA could form H-bonds with Arg105 and aromatic interactions with Arg66 in the endogenous ligand’s active site of Bcl-2 (4IEH), while ADM formed H-bonds with Ala59, Arg66, Asn102, and Try161, H-bonds plus aromatic interactions with Gly104, and Arg105 (Fig.7). Tanshinone IIA could form H-bonds with Lys890 and aromatic interactions with Met953 in the endogenous ligand’s active site of PI3K (4J6I), while ADM could only form H-bonds with Val882, Ala885, Asp884, Thr887, Lys890, Asp950, and Met953 (Fig. 8). Only tanshinone IIA could form H-bonds with Cys919, aromatic interactions with Leu840 and Val848 in the endogenous ligand’s active site of VEGFR-2 (3VHE) (Fig. 9). Thus, these results indicated that tanshinone IIA may display the anti-tumor effect with similar molecular mechanisms of ADM: to interact with the proteins in the same active sites, but to interact with different residues.

## Discussion

In this study, we found that the combination of tanshinone IIA with ADM could suppress cell proliferation, induce apoptosis, and impair cell repair motility and migration ability in A549 cells with a synergistic manner. In addition, our findings indicated that the potential pro-apoptotic mechanism of tanshinone IIA and ADM on A549 cell lines may involved the suppression in VEGF/PI3K/Akt pathway.

Based on the IC_50_ values of the CCK8 assay and our preliminary study results [12], we demonstrated that both tanshinone IIA and ADM had inhibitory effect on proliferation of A549, PC9, and HLF cells in a dose-dependent and time-dependent manner. Compared with ADM, the inhibitory effect of tanshinone IIA was much weaker on the tested cell lines. Different from the two NSCLC cell lines, HLF cells displayed a stronger sensitivity to ADM, while a much weaker sensitivity to tanshinone IIA, which indicated that tanshinone IIA might have little toxicity to human normal cells, with ADM showing the opposite effect. As for the drug combination, it exerted synergistic inhibitory effects in A549 cells and PC9 cells, while antagonism effects in HLF cells. Based on these, we hypothesized that tanshinone IIA might potentiate the sensibility of chemotherapy for NSCLC patients while minimizing harm to normal cells.

Our current study also provides insight into the mechanism of the synergistic effect of ADM in combination with tanshinone IIA on apoptosis and cell cycle distribution by TUNEL and FCM experiments in A549 cells. However, it is worth to mention that in FCM results, cells in the lower left quadrant moved to the upper left quadrant in both ADM groups and drug combination groups. This may due to the similar color excitation wavelength of PI and the autofluorescence of ADM.

FCM showed that both drug combination treatment and single ADM treatment caused G2 phase arrest in A549 cells, while single tanshinone IIA treatment caused S phase arrest. Cell population of G1 phase was decreased in all drug treatment groups, with drug combination groups showing the statistically significant deduction, which was consistent with many other researchers’ findings. Tung’s study found that in the human lung adenocarcinoma cell lines (A549, CL1-0, and CL1-5), tanshinone IIA was likely to slow the progression of S to G2 phases of the cell cycle [17]. The similar results were found in the renal cancer cell line 786-O cells, the percentage of cells in S phase was increased in a dose-dependent manner with the tanshinone IIA treatment (0, 6.79, 13.59 or 27.18 μM, 24 h) [18]. Wang’s research team found that co-treatment of ADM (0.75 μM, 48 h) and itraconazole (6 μM, 48 h) brought about a notable increase in G2 phase and a decrease in G1 and S phase in the acute myeloid leukemia cells KG1α [19]. However, the effect of tanshinone IIA on the cell cycle distribution is still controversial. Ma found that in NSCLC cell line H1299 cells, a proportion of cells at the G1 phase increased compared with the control when treated with tanshinone IIA (4 μM, 48 h) [20].

These findings showed that low dosage tanshinone IIA might contribute to the cell cycle arrest at G1 phase, while high dosage tanshinone IIA might lead to an S phase’s cell cycle blockage in NSCLC cell lines, which remains to be further explored.

Meanwhile, the TUNEL assay and Cleaved Caspase-3 immunofluorescence staining results confirmed that tanshinone IIA and ADM induced cell apoptosis by causing DNA damage and increasing the expression of the pro-apoptotic protein Cleaved Caspase-3 expression. We next explored the possible pathway related to this protein.

As we all known, VEGF is one of the most significant and specific angiogenesis factors [21], and is a potent angiogenic catalyst secreted by many types of tumor cells. VEGF family members bind to the three overlapping VEGFRs, which are activated by their cognate ligands and modulated by a variety of biological processes including dimerization, internalization, degradation, and receptor presentation [22, 23]. Notably, VEGFR2 plays a key role in the VEGF/VEGFR2 pathway in regards to angiogenesis and tumor growth [24, 25]. Recent researches show that VEGF regulates VEGFR2 by forming directly physical interaction with VEGFR2 [23, 26–31] and/or induces the phosphorylation of VEGFR2 [27–32]. There are many studies related to downstream targets of VEGFR2, such as the PI3K/Akt pathway [33].

The phosphatidylinositol 3-kinase (PI3K)/Akt pathway is a cascade of events, which plays a critical role in a broad variety of pathophysiological processes. It is well established that the pathway is one of the most important oncogenic pathways in almost all kinds of human cancers [34], being vital to growth and survival of cancer cells [35–38], as well as disease initiation and development, including tumorigenesis, proliferation, invasion, cell cycle progression, inhibition of apoptosis, angiogenesis, metastasis and chemoresistance in cancer cells [39].

PI3-kinase is activated by a variety of growth factors binding to their receptors. Such as fibroblast growth factors, epidermal growth factor, VEGF, hepatocyte growth factor, IL-8 and so on, which are known to induce angiogenesis [40, 41]. As PI3K/Akt signaling becomes a major downstream intracellular pathway that mediate the biological effects of VEGF [42], a substantial number of studies have been conducted to support the important role of VEGF/PI3K/Akt signaling in tumor progression [43–47].

Our western blot results revealed that the drugs treatment gave rise to distinctly high expressions of Cleaved-Caspase-3 and Bax, but low expressions of VEGF, VEGFR2, Bcl-2, Caspase-3, p-PI3K and p-Akt proteins, with total PI3K and Akt proteins expression remaining nearly the same. The effects of the drug combination treatment showed more significant difference compared with single drug treatment.

In light of this, we hypothesized that drugs induced pro-apoptotic process was likely associated with the down-regulation of the VEGF/PI3K/Akt signaling pathway. Bax and Cleaved Caspase-3 were up-regulated, suggesting that the mitochondrial apoptotic pathway [48–50] was also involved in Tan IIA-induced A549 cell death, which are consistent with many other scientists’ research [33, 51, 52].

These findings showed some similarities with some recent studies by other scientists [53–56]. Li found that tanshinone IIA could inhibit the angiogenesis and growth of human breast cancer xenografts in nude mice and inhibit VEGF expression in breast cancer cells via mTOR/p70S6K/RPS6/4E-BP1 signaling pathway. Xing found that tanshinone IIA had a negative effect on cell proliferation, migration and tube formation of human umbilical vascular endothelial cells (HUVEC) via the simultaneous inhibition of the VEGF/VEGFR2 signaling pathway.

However, there are some different outcomes of tanshinone IIA’s effect on VEGF expression. Xu found that tanshinone IIA could promote angiogenesis and up-regulate VEGF expression in myocardial infarction (MI) rats [57]. Furthermore, Xu found that the expression of VEGF was up-regulated in tanshinone IIA-pretreated flap tissue [58]. We proposed that this might be related to the protective function of tanshinone IIA in myocardial cells and epithelial skin cells, which is opposite to cancer cells and HUVEC. Therefore, the effect of tanshinone IIA on A549 cells and the underlying mechanisms need to be further explored.

Furthermore, there are many newly reported researches emphasize on the crucial role of the target protein VEGFR2 in the VEGF/VEGFR2 mediated PI3K/Akt signal pathway verified by the VEGFR2, PI3K and Akt inhibitors [28–31]. However, some scientists thought PI3K may also play an important role in some occasions [59–63]. These studies provide deeper research clues and theoretical support for our current research results.

**Fig. 10:**
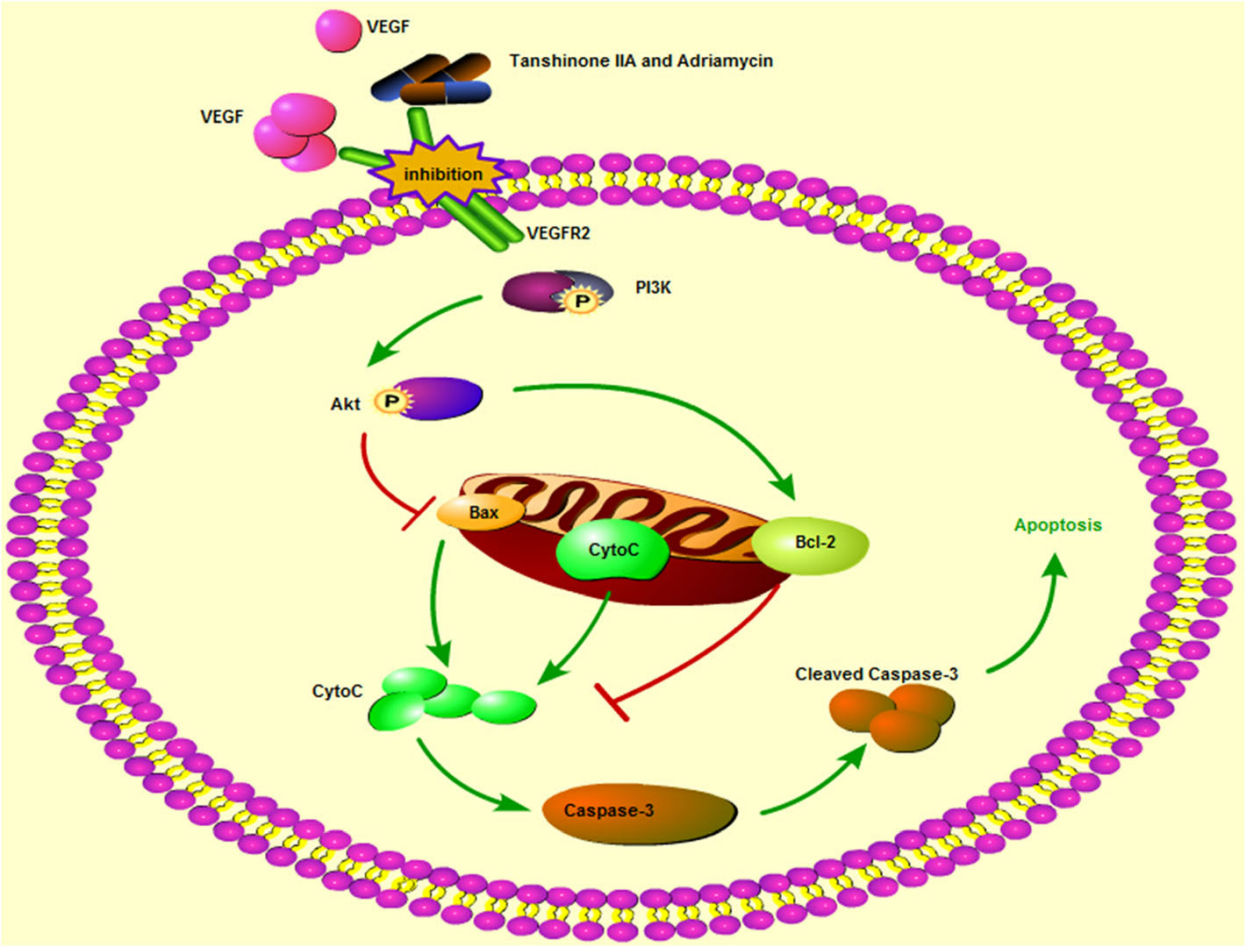
The proposed signal transduction pathway caused by tanshinone IIA and ADM in A549 cells

Considering our previous study [12] along with the research mentioned above [48], we speculated that tanshinone IIA might act as a competitive inhibitor of VEGFR2, forming complexes with VEGFR2 to interdict the binding of VEGF to VEGFR2 and the phosphorylation of VEGFR2, sequentially inhibited tumour angiogenesis, cell migration and tumorigenicity [64]. Combined with the molecular docking analysis results, tanshinone IIA and ADM could also target the protein kinase domains of Akt2 (PDB-ID: 2JDR), Bcl2 (PDB-ID: 4IEH) and PI3K (PDB-ID: 4J6I), which may contribute to the inhibition of the downstream PI3K/Akt signal pathway, which interrupts the phosphorylation of PI3K and Akt, causing the apoptosis of A549 cells. Besides, the inactivated Akt could not phosphorylate the pro-apoptotic proteins such as Bad, Bax and caspase families, therefore losing the function of inhibiting these pro-apoptotic proteins. The activated Caspase-3 cascade triggers the mitochondrial-induced apoptotic pathway, with the release of cytochrome c from the mitochondria and Bax translocation to the mitochondria, causing an increasing ratio of Bax/Bcl-2. The mitochondrial apoptotic process produced both initiators and effectors of the apoptotic programmed cell death (Fig. 10).

Therefore, we might conclude that tanshinone IIA and ADM suppressed the VEGF/PI3K/Akt pathway, induced mitochondrial dysfunction, triggered the mitochondrial-induced apoptotic pathway and then gave rise to the cell apoptosis. Meanwhile, the drugs combination groups showed synergistic effects in the pro-apoptotic process.

The advantages of methodology, clinical application prospect: this is the first report on the effect of tanshinone IIA in combination with ADM on NSCLC A549 Cell Line. This study established an objective evaluation system for the efficacy of extracts of Chinese herbal medicines and chemotherapy drugs used in combination, and explored its underlying mechanisms. In this study, we found that tanshinone IIA combined with ADM presented synergistic effects on proliferation suppression in series of cancer cells, but not in normal cells. The mechanisms might be through the down-regulation of VEGF/PI3K/Akt pathway and inducing the mitochondria dependent apoptosis.

These investigations indicated that the inhibition of the VEGF/PI3K/Akt signaling cascade could be served as an effective strategy for the treatment of cancers. Agents (such as tanshinone IIA) targeting the apoptosis pathway without affecting normal cells might play crucial roles as potential drug targets in cancer management, which will minimize adverse effects, maximize clinical outcomes, and help improve the patients’ quality of life.

The existing problems and future study directions: to provide further evidence to allow in-depth understanding of the synergistic anti-cancer activity of tanshinone IIA and ADM. Further study on the probable molecular mechanisms in VEGF/PI3K/Akt signal pathway need to be carried out with antagonist antibodies, and the synergistic anti-cancer activity in vivo should be verified. Based on the results of this study we believe tanshinone IIA should be developed further as a possible new therapeutic adjuvant treatment for NSCLC.

## Conclusions

Our findings indicated that the combination of tanshinone IIA with ADM could inhibit malignant biological behaviors of NSCLC cell line in a synergistic way. Tanshinone IIA could be used as a novel agent in postoperative adjuvant therapy and improve the sensibility of chemotherapeutics for NSCLC with less side effects. In addition, this experiment could not only provide a reference for the development of more effective anti-tumor medicines, but also build a platform for evaluating the effects of herbs and chemotherapy drugs used in combination.

## Additional file

Additional file 1: Inhibitive effects of Tanshinone IIA and Adriamycin on A549, PC9 and HLF cells. A549 (A), PC9 (C) and HLF (E) cells were treated with 8 μM, 4 μM, 2 μM, 1 μM, 0.5 μM, 0.25 μM, 0 μM of Adriamycin for 24 h, 48 h and 72 h respectively. At the same time, A549 (B), PC9 (D) and HLF (F) cells were treated with 320 μM, 160 μM, 80 μM, 40 μM, 20 μM, 10 μM, 5 μM, 0 μM of Tanshinone IIA for 24 h, 48 h and 72 h respectively. Data were derived from three independent experiments. (TIF 747 kb)

## Abbreviations

ADM: Adriamycin
AF: Alexa Fluor
Akt: Protein kinase B
Ala: Alanine
Arg: Arginine
Asn: Asparagine
Asp: Asparagine acid
CI: Combination index
CID: Compound ID
Cys: Cysteine
DMSO: Dimethylsulfoxide
ECL: Enhanced chemiluminescence
FBS: Fetal bovine serum
FCM: Flow cytometry
Glu: Glutamic acid
Gly: Glycine
HRP: Horseradish peroxidase
IC_50_: 50% inhibitory concentration
Leu: Leucine
Lys: Lysine
Met: Methionine
mTOR: mammalian target of rapamycin
NS: Normal saline
NSCLC: Non-small cell lung cancer
Phe: Phenylalanine
PI3K: Phosphatidylinositol 3-kinase
PVDF: Polyvinylidene fluoride
RTKs: Receptor tyrosine kinases
Tan I: Tanshinone I
Tan IIA: Tanshinone IIA
TBST: Tris Buffered Saline with Tween
TCM: Traditional Chinese medicine
Thr: Threonine
Try: Tryptophan
TUNEL: Terminal-deoxynucleoitidylTransferase Mediated Nick End Labeling
Val: Valine
