# Supplementary figures and images for: Tanshinone IIA combined with adriamycin inhibited malignant biological behaviors of NSCLC A549 cell line in a synergistic way
Source: BMC Cancer. 2016 Nov 18;16:899. doi: 10.1186/s12885-016-2921-x (PMC5116215; doi:10.1186/s12885-016-2921-x)

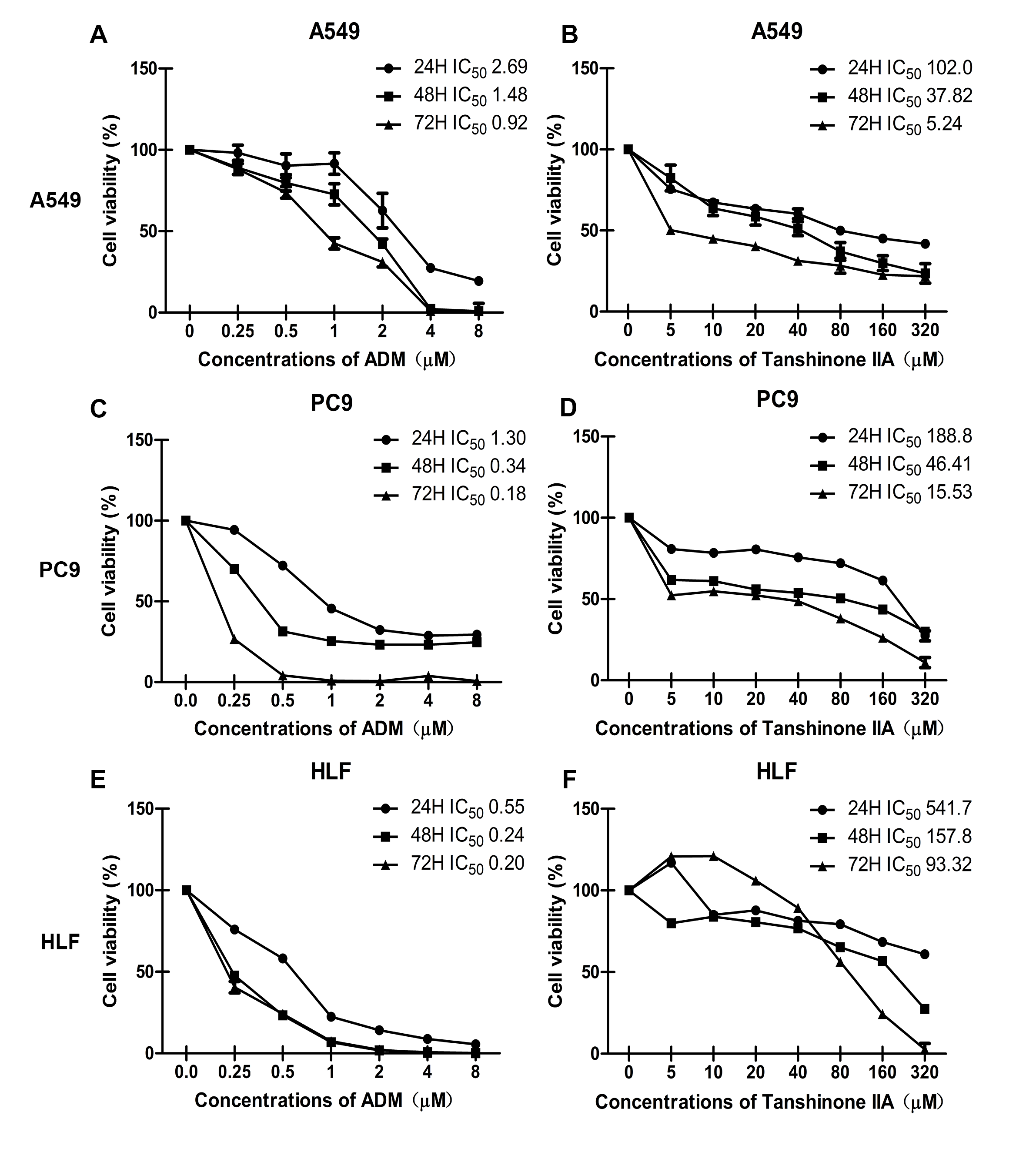

Supplement: Additional file 1: — Inhibitive effects of Tanshinone IIA and Adriamycin on A549, PC9 and HLF cells. A549 (A), PC9 (C) and HLF (E) cells were treated with 8 μM, 4 μM, 2 μM, 1 μM, 0.5 μM, 0.25 μM, 0 μM of Adriamycin for 24 h, 48 h and 72 h respectively. At the same time, A549 (B), PC9 (D) and HLF (F) cells were treated with 320 μM, 160 μM, 80 μM, 40 μM, 20 μM, 10 μM, 5 μM, 0 μM of Tanshinone IIA for 24 h, 48 h and 72 h respectively. Data were derived from three independent experiments. (TIF 747 kb) [file 12885_2016_2921_MOESM1_ESM.tif]
